# Vaccinia-related kinase 1 promotes hepatocellular carcinoma by controlling the levels of cell cycle regulators associated with G1/S transition

**DOI:** 10.18632/oncotarget.4967

**Published:** 2015-09-07

**Authors:** Namgyu Lee, Jung-Hee Kwon, Young Bae Kim, Seong-Hoon Kim, Sung Jin Park, Weiguang Xu, Hoe-Yune Jung, Kyong-Tai Kim, Hee Jung Wang, Kwan Yong Choi

**Affiliations:** ^1^ Department of Life Sciences, Pohang University of Science and Technology, Pohang, Gyeongbuk, Korea; ^2^ Cbs Bioscience Inc., Daejeon, Korea; ^3^ Department of Surgery, Ajou University School of Medicine, Suwon, Korea; ^4^ Department of Pathology, Ajou University School of Medicine, Suwon, Korea; ^5^ Department of Integrative Biosciences & Biotechnology, Pohang University of Science and Technology, Pohang, Gyeongbuk, Republic of Korea

**Keywords:** HCC, VRK1, proliferation, cell cycle, luteolin

## Abstract

We identified the specific role of vaccinia-related kinase 1 (VRK1) in the progression of hepatocellular carcinoma (HCC) and evaluated its therapeutic and prognostic potential. VRK1 levels were significantly higher in HCC cell lines than a normal hepatic cell line, and were higher in HCC than non-tumor tissue. VRK1 knockdown inhibited the proliferation of SK-Hep1, SH-J1 and Hep3B cells; moreover, depletion of VRK1 suppressed HCC tumor growth *in vivo*. We also showed that VRK1 knockdown increased the number of G1 arrested cells by decreasing cyclin D1 and p-Rb while upregulating p21 and p27, and that VRK1 depletion downregulated phosphorylation of CREB, a transcription factor regulating CCND1. Additionally, we found that luteolin, a VRK1 inhibitor, suppressed HCC growth *in vitro* and *in vivo*, and that the aberrant VRK1 expression correlated with poor prognostic features of HCC. High levels of VRK1 were associated with shorter overall and disease-free survival and higher recurrence rates. Taken together, our findings suggest VRK1 may act as a tumor promoter by controlling the level of cell cycle regulators associated with G1/S transition and could potentially serve as a therapeutic target and/or prognostic biomarker for HCC.

## INTRODUCTION

Liver cancer is the second leading cause of cancer-related death among males and the sixth most common cause of death among females, worldwide [1]. Hepatocellular carcinoma (HCC) accounts for 85% to 90% primary liver cancers [2]. Although therapeutic options for HCC have improved in recent years, incidences of the disease have remained high in Eastern and South-Eastern Asia and are increasing in the United States and Central Europe [1]. The major reason for unsuccessful treatment of HCC is resistance to conventional chemotherapy [3], and surgical resection and liver transplantation have limited applicability due to frequent tumor recurrence [4]. Undoubtedly, a better understanding of the mechanisms underlying HCC progression is crucial for effective treatment of the disease.

Vaccinia-related kinase 1 (VRK1) is a member of the Ser/Thr kinase family in mammals [3], and is involved in cell cycle progression, chromosome condensation, nuclear envelope breakdown and reassembly and the DNA damage response. VRK1 acts through phosphorylation of several substrates, including CRE binding protein, histone H3, barrier-to-autointegration factor and p53 [4–9]. VRK1 mediates p53 accumulation by increasing its stability through phosphorylation of Thr-18 within its mdm-2 binding site [10, 11]. At the same time, VRK1 levels are downregulated by p53, forming autoregulatory loop [12]. This p53-induced downregulation of VRK1 is dependent on an autophagic pathway and protein degradation by lysosomes [13].

Emerging evidence suggests VRK1 plays an essential role in cancer progression [3]. For example, high levels of VRK1 mRNA have been detected in actively proliferating cells within fetal tissues and in several cancer cell lines [14]. In addition, VRK1 expression correlates positively with several proliferation markers in head-and-neck squamous cell cancers and lung carcinomas [15, 16], and VRK1 levels tend to be elevated in lung cancer tissues in which p53 is mutated [16]. In breast cancer, VRK1 depletion inhibits tumor growth and metastasis [17] and confers resistance to DNA-damaging agents [18], and it has been suggested that VRK1 is a potential therapeutic target and a prognostic marker for breast cancer [19, 20].

On the other hand, there have been few studies examining the precise role of VRK1 in the progression of HCC or its clinical association with HCC. In the present study, therefore, our aim was to determine the function of VRK1 within HCC tissues and cell lines. Our findings suggest that VRK1 enhances HCC cell proliferation by modulating the levels of regulators associated with G1/S transition and that VRK1 levels are much higher in HCC tissues than non-tumor tissues, and are associated with shorter overall and disease-free survival and a higher recurrence rate. Based on these findings, we propose that VRK1 could potentially serve as a therapeutic target and/or a prognostic marker in HCC.

## RESULTS

### VRK1 is overexpressed in HCC cells and its depletion suppresses HCC cell proliferation *in vitro*

**Figure 1:**
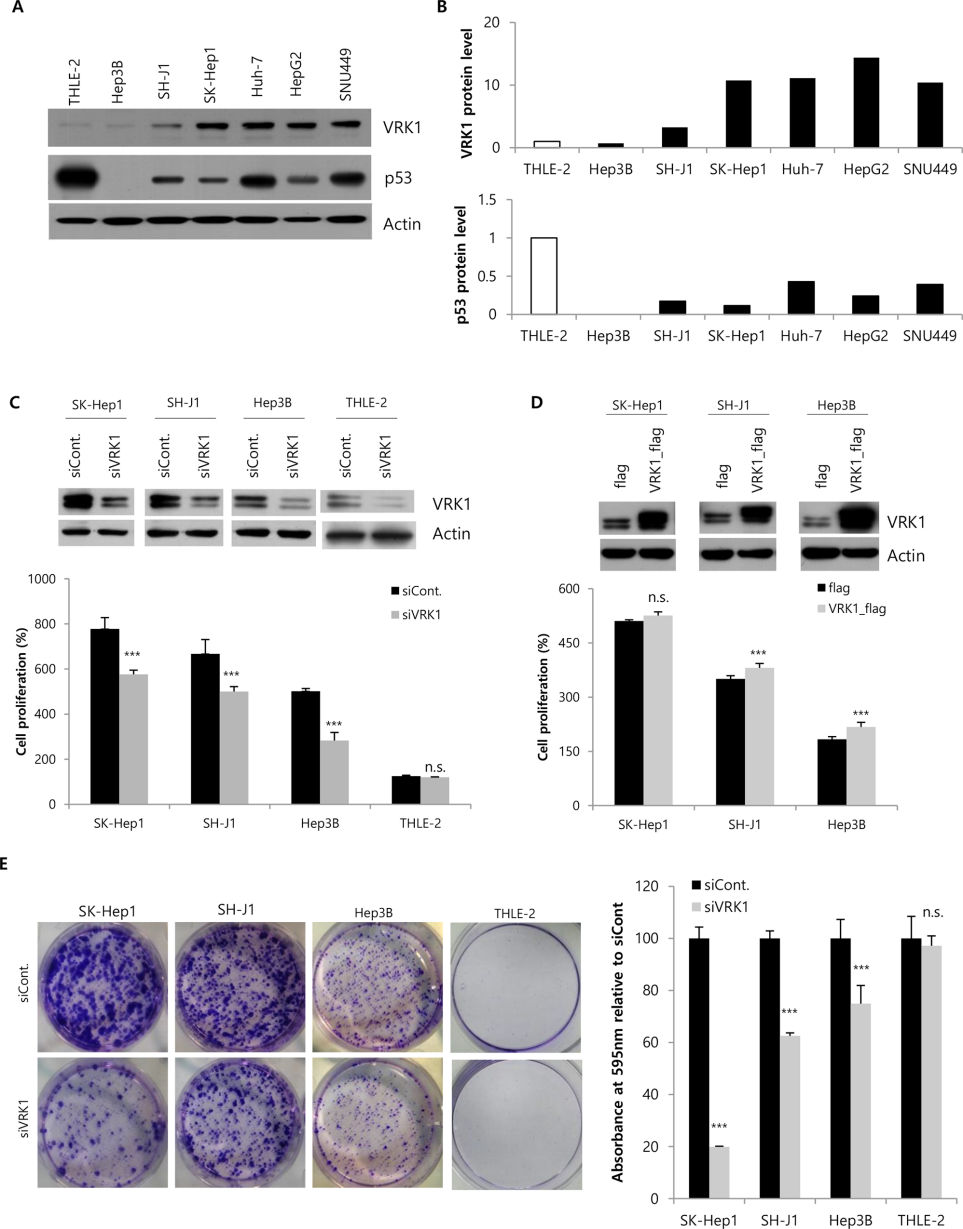
Regulation of HCC cell growth by VRK1 *in vitro* **A.** The levels of VRK1 in THLE-2 cells, an immortalized normal cell line, and six HCC cell lines were analyzed by Western blotting using actin as a loading control. **B.** Band intensities were measured by densitometry using Image J. VRK1 and p53 band intensities were normalized to those of actin. **C.** VRK1 levels were reduced in HCC and THLE-2 cells 48 h after transfection with VRK1 siRNA as judged by Western blot analysis. Cell proliferation after silencing VRK1 expression was assessed with WST-1 assays 72 h after transfection. Cell proliferation (%) were calculated relative to that at the time of transfection, which was set to 100%. **D.** Cells were transfected with flag- or VRK1-flag-expressing vectors, and VRK1 level was analyzed by Western blotting 48 h after transfection. Increases of cell proliferation were significant in SH-J1 and Hep3B cells upon overexpression of VRK1, but not in SK-Hep1 cells. **E.** Colony formation assays were performed using HCC or THLE-2 cells transfected with control siRNA or VRK1 siRNA. Magnified image of a THLE-2 cell colony is shown in Supplementary Figure 2. Cells were grown for 10 days and stained with crystal violet (Left). Colonies were quantified by measuring the absorbance of extracted crystal violet at 595 nm (Right). (***p < 0.001; n.s., not significant)

To identify the role of VRK1 in liver cancer, VRK1 levels were examined in an immortalized hepatocyte cell line, THLE-2, and in six HCC cell lines, including SH-J1, SK-Hep1, Huh-7, Hep3B, HepG2 and SNU449. With the exception of Hep3B cells, which grow slower the other HCC cells, VRK1 levels were higher in HCC cells than THLE-2 cells (Fig. 1A and 1B). VRK1 expression is known to be enhanced in lung cancers expressing a mutant p53 and to be down-regulated by ectopic expression of wild-type p53 in lung cancer cells [16]. Therefore, to investigate why VRK1 levels are higher in HCC cells, we checked the levels and status of p53. p53 expression was relatively high in THLE-2, Huh-7 and SNU449 cells, but low in SH-J1, SK-Hep1, HepG2 and Hep3B cells (Fig. 1A and 1B). We found that there was not a detectable inverse correlation between p53 and VRK1 levels in the seven cell lines. We then examined VRK1 levels after transfecting SK-Hep1, SH-J1 and Hep3B cells with increasing amounts of pcDNA_p53 and the same amount of pCMV_VRK1-flag (Sup. 1A and 1B). As the level of p53 increased, the level of VRK1 expression declined in these cells (Sup. 1A and 1B). Thus an inverse relation was detected between the levels of VRK1 and ectopically expressed p53 (Sup. 1A and 1B).

The status of p53 in the tested HCC cell lines was examined previously and those findings are summarized in Table 1. p53 is wild-type in THLE-2, SH-J1, SK-Hep1 and HepG2 cells, but is mutated in Huh-7 and SNU449 cells, and null in Hep3 cells. As with p53 expression levels, there was no correlation between the status of p53 and VRK1 levels in any of the seven cell lines (Fig. 1A and 1B). Additionally, because HBV infection is a major risk factor for HCC development, the association between HBV status and VRK1 expression was assessed in the seven cell lines, several of which are HBV-positive (Table 1). However, we found no significant correlation between VRK1 expression and HBV status.

**Table 1:** HBV and p53 status of HCC cells

	HBV	p53
THLE-2	Immortalized normal cell	
SK-Hep1	Negative [45]	WT [11]
SH-J1	Negative [37]	WT [46]
Hep3B	Positive [45]	Null [11, 47]
Huh-7	Negative [48]	Mutated [11]
HepG2	Negative [45]	WT [11]
SNU449	Positive [49]	Mutated [11]

**Figure 2:**
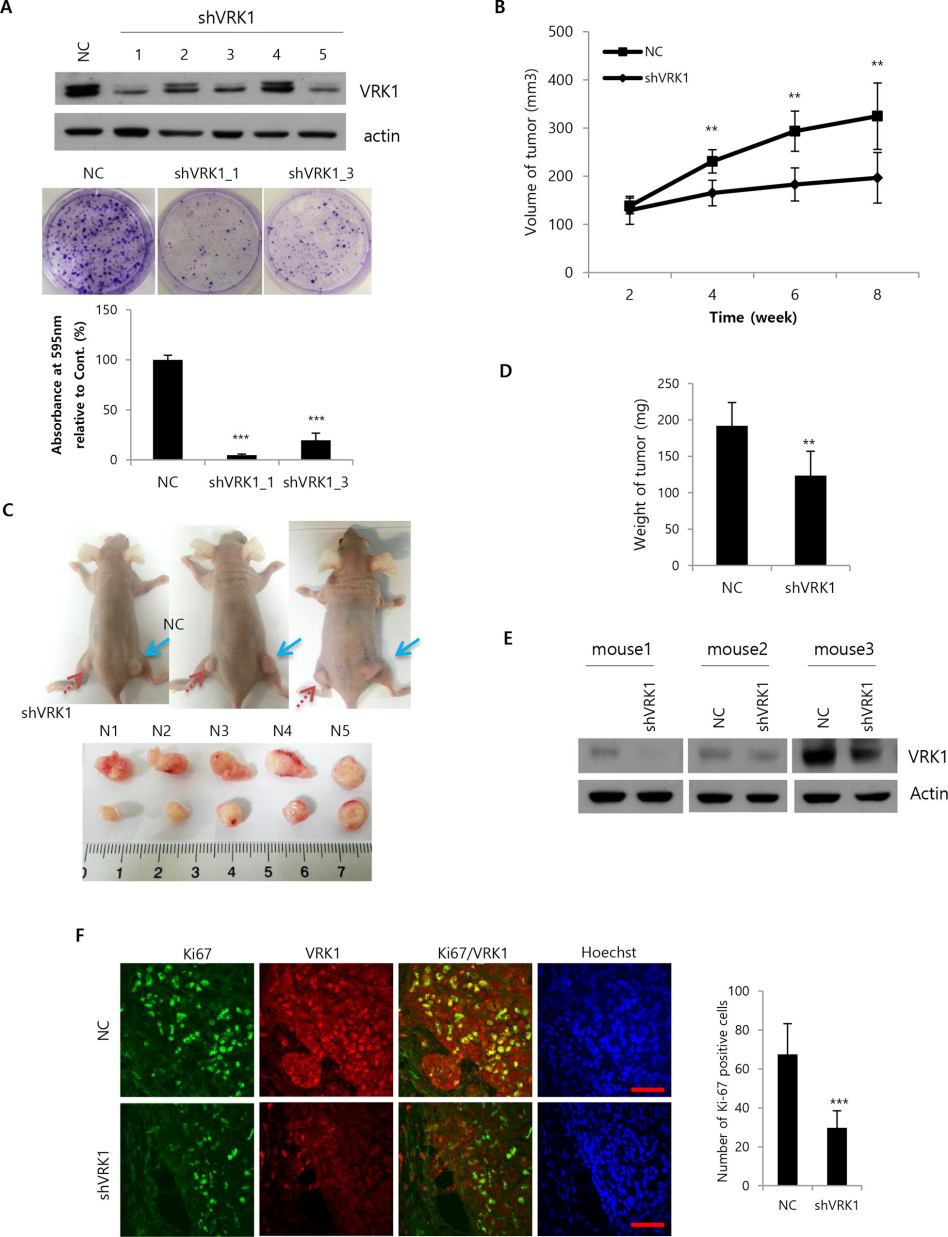
Growth of HCC tumors after VRK1 depletion *in vivo* **A.** SK-Hep1 cells were transduced with a lentiviral vector encoding VRK1- or negative control (NC)-shRNA sequences. Five lentiviral particles (Clones 1–5) targeted to different sequences in the VRK1 gene were used and the efficiencies of the VRK1 depletion were compared by Western blotting 1 week after transduction (upper panel). Colony formation assays were performed using stably transduced SK-Hep1 cells (middle panel). Colonies were quantified by measuring the absorbance of extracted crystal violet at 595 nm (lower panel). **B.** 5 × 10^6^ cells were injected subcutaneously. Tumor sizes were measured in 10 mice every 2 weeks after injection, and are shown as means ± SEM (upper panel). **C.** Photographs of mice were taken after sacrifice. NC (blue arrow) and shVRK1 (red arrow) indicate the flanks injected with SK-Hep1 cells transduced with NC shRNA or VRK1 shRNA (upper panel). Tumors were collected from the sacrificed mice and their sizes compared (lower panel). **D.** Tumor weights were measured after sacrifice of mice, and are shown as means ± SEM. **E.** Levels of VRK1 in injected tumors were analyzed by Western blotting after sacrifice. **F.** Levels of Ki-67 and VRK1 were assessed immunohistochemically using a confocal microscope, and Ki-67-positive cells were counted in randomly obtained images. Scale bar indicates 50 μm. (***p < 0.001; **p < 0.01)

To better understand the molecular function of VRK1 in HCC tumorigenesis, we transiently silenced VRK1 expression or overexpressed VRK1 in THLE-2 and HCC cell lines, after which we measured cell proliferation using WST-1 assays. Western blotting showed that VRK1 was efficiently knocked down or overexpressed by transfection of a targeted siRNA or expression vector, respectively (Fig. 1C and 1D, upper panel). Significant reductions in proliferation were observed in HCC cell lines following VRK1 knockdown (*P* < 0.001), but not in THLE-2 cells (Fig. 1C, lower panel). Conversely, overexpression of VRK1 significantly enhanced the proliferation of SH-J1 and Hep3B cells (*P* < 0.001), but not SK-Hep1 cells (Fig. 1D, lower panel). To further evaluate the long-term effects of reducing VRK1 expression on cellular proliferation, we performed as set of colony formation assays (Fig. 2E). Knocking down VRK1 expression reduced the size of all HCC cell colonies (*P* < 0.001), with the most dramatic effect on SK-Hep1 cells, which normally express the highest level of VRK1 (19.86% ± 0.27), and the smallest effect on Hep3B cells, which normally express the lowest levels of VRK1 (74.92% ± 6.98; Fig. 2E). By contrast, knocking down VRK1 had no significant effect on the size of THLE-2 cell colonies (97.16 ± 3.81; Fig. 2E and Sup. Fig. 2).

### VRK1 depletion inhibits tumor growth in a xenograft mouse model

To investigate the contribution of VRK1 to tumor growth *in vivo*, we established SK-Hep1 cells expressing shRNA targeting VRK1 through transduction using a lentiviral vector. Two weeks after transduction, different knockdown efficiencies were observed with for independent shRNAs targeting different sequences of VRK1 (Fig. 2A, upper panel). Colony formation assays with stable cell lines (clones 1 and 3) showed efficient VRK1 knockdown and confirmed significant loss of colony forming ability (*P* < 0.001; Fig. 2A, middle and lower panel). Clone 1 expressing the lowest level of VRK1 displayed the most dramatic decrease in colony formation (4.73% ± 1.02, Fig. 2A, lower panel). After 3, 4, 5 and 6 weeks of viral transduction, stable cell lines were subjected to Western blot analysis and colony formation assays to confirm the anti-tumor effect by sustained VRK1 knockdown. Efficient knockdown and diminished colony formation were maintained in stable VRK1-deficient cells for least 6 weeks (Sup. Fig. 3A and 3B).

Once the stability of the VRK1 knockdown was confirmed, we injected cell lines stably expressing VRK1 shRNA Clone 1 into the right flanks of nude mice and negative control shRNA into the left flanks. Tumor volumes were then determined every 2 weeks. Significant differences in volume between tumors expressing shVRK1 and those expressing control shRNA were observed beginning 4 weeks after injection (*P* < 0.01; Fig. 2B), and at 8 weeks the mean volume of shVRK1-expressing tumors was 196.67 ± 52.40 mm^3^, while that of tumors expressing control shRNA was 324.61 ± 68.95 mm^3^ (Fig. 2B and 2C). In addition, the weights of shVRK1-expressing tumors were correspondingly lower than the weights of tumors expressing control shRNA (111.67 ± 21.08 mg vs. 164.17 ± 37.17 mg; Fig. 2D).

To confirm the efficiency of the sustained VRK1 knockdown during tumor growth *in vivo*, Western blotting was performed using tumor tissues collected from each group of mice. Significant downregulation of VRK1 was observed in the tumor tissues derived from VRK1 knockdown cells, as expected (Fig. 2E). Additionally, immunohistochemical staining for Ki-67, a general marker of proliferating cells, showed significantly fewer Ki-67-positive cells in tumors expressing shVRK1 (29.75 cells ± 78) than in those expressing negative control shRNA (67.5 cells ± 15.77; Fig. 2F).

### VRK1 depletion increases the G1 arrest in HCC cells by controlling the level of cell cycle regulators

**Figure 3:**
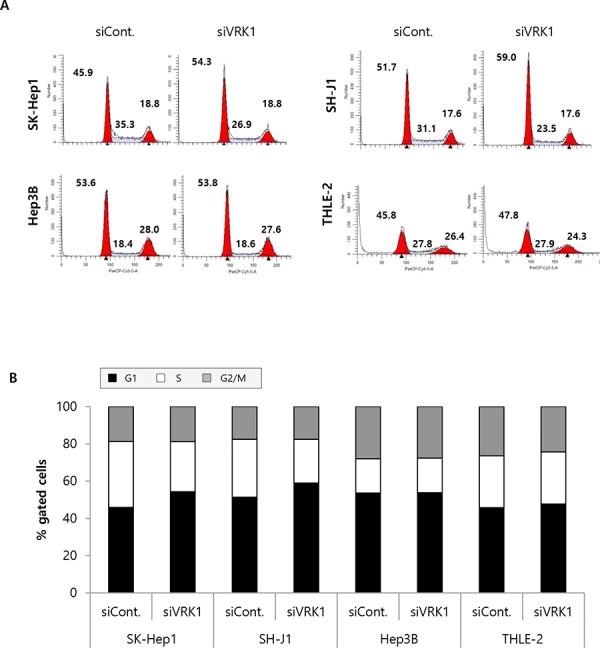

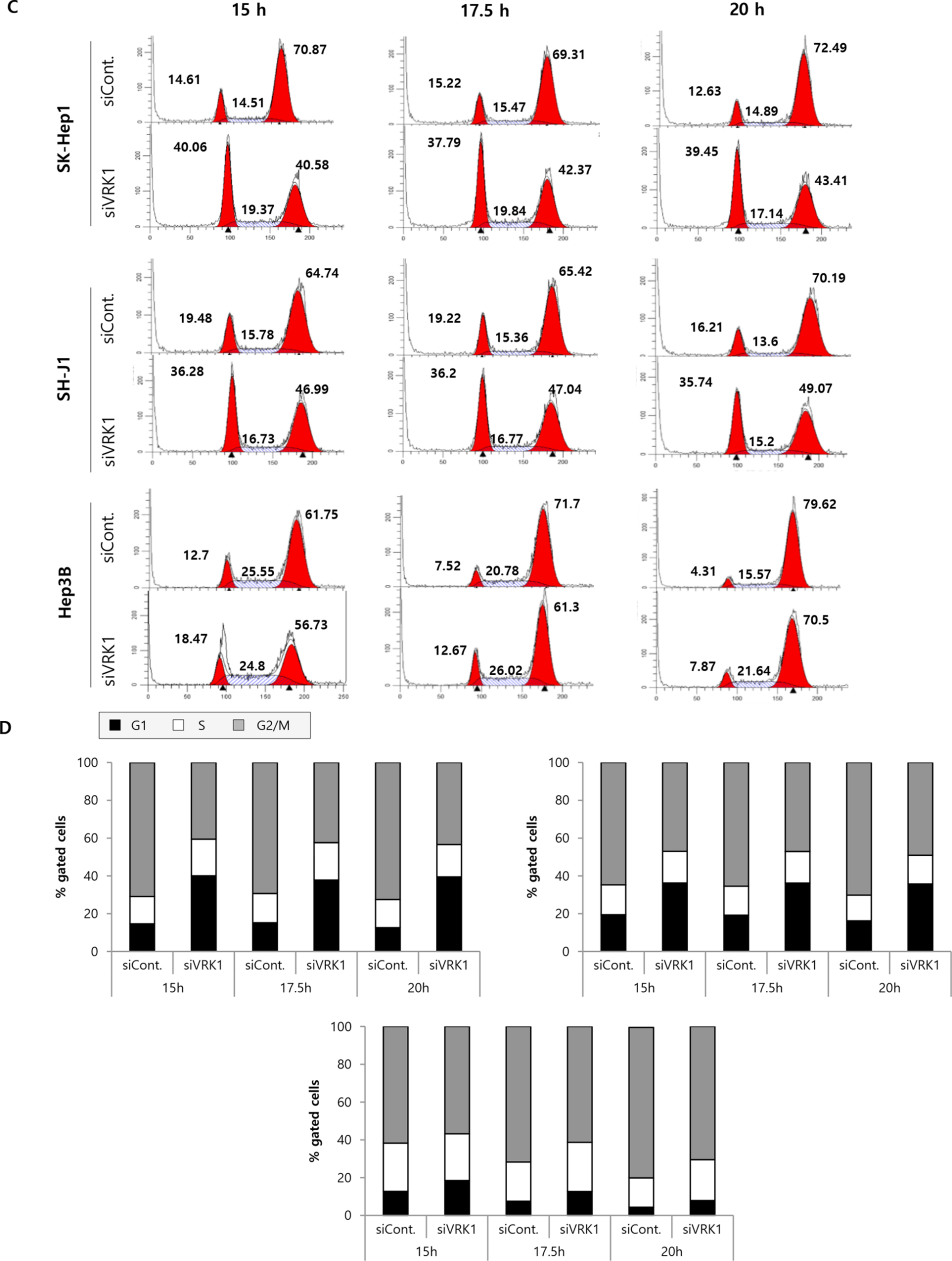
FACS analysis of VRK1-depleted HCC cells **A.** SK-Hep1, SH-J1, Hep3B and THLE-2 cells were transfected with control or VRK1 siRNAs and grown for 24 h, after which they were stained with propidium iodide (PI) and subjected to FACS analysis. The number of HCC cells at G1, S and G2/M phase was quantified. The Modifit program was used for data analysis. **B.** The percentages of cells at G1, S and G2/M phase are indicated in the histogram. **C.** Cells were incubated with 50 ng/ml nocodazole 1 day after transfection. After the indicated incubation times (15 h, 17.5 h and 20 h) with nocodazole, the cells were stained with PI and subjected to FACS analysis. **D.** The percentages of cells treated with nocodazole at G1, S and G2/M phase are indicated in the histogram. Three independent experiments were performed and representative FACS data are shown.

Anti-growth effects are generally explained as the result of cell cycle disruption and/or apoptosis or senescence induction. We explored the effects of VRK1 depletion on induction of apoptosis and cell cycle regulation. Induction of apoptosis was measured using the PI-Annexin V double staining method 24 h after transfection (Sup. 4A and 4B). No significant induction of apoptosis was detected in HCC cells following depletion of VRK1 (Sup. 4A and 4B). On the other hand, 24 h after transfection with VRK1 siRNA, the numbers of G1 phase gated cells were significantly higher in SK-Hep1 and SH-J1 than control cells, whereas the numbers of S phase gated cells were lower (Fig. 3A and 3B). By contrast, no significant changes in cell cycle populations were found in Hep3B and THLE-2 cells after VRK1 depletion in an asynchronous cell population (Fig. 3A and 3B). Nocodazole arrests cell cycle progression in G2/M phase by disrupting mitotic spindles [21], and so was used to assess G1 arrest induced by VRK1 depletion. Following nocodazole treatment, the majority of control cells were synchronized at G2/M phase, whereas VRK1-depleted cells could not be synchronized at G2/M phase at any time during the time-course of the experiment (Fig. 3C and 3D). Instead, most VRK1-depleted cells were at G1 phase (Fig. 3C and 3D). The largest G1 phase gated cell fraction was among VRK1-depleted SK-Hep1 cells (40.06% at 15 h, 37.79% at 17.5 h, 39.45% at 20 h; Fig. 3C and 3D), followed by VRK1-depleted SH-J1 cells (36.28% at 15 h, 36.20% at 17.5 h, 35.74% at 20 h; Fig. 3C and 3D). The smallest G1 phase gated cell fraction was among VRK1-depleted Hep3B cells (18.47% at 15 h, 12.67 at 17.5 h, 7.84% at 20 h; Fig. 3C and 3D). Following nocodazole treatment, the size of the G1 phase gated cell fraction remained unchanged over the observation time-course in VRK1-depleted SK-Hep1 and SH-J1 cells, but it gradually declined in VRK1-depleted Hep3B cells (18.47% at 15 h, 12.67% at 17.5 h and 7.87% at 20 h; 3C and 3D).

**Figure 4:**
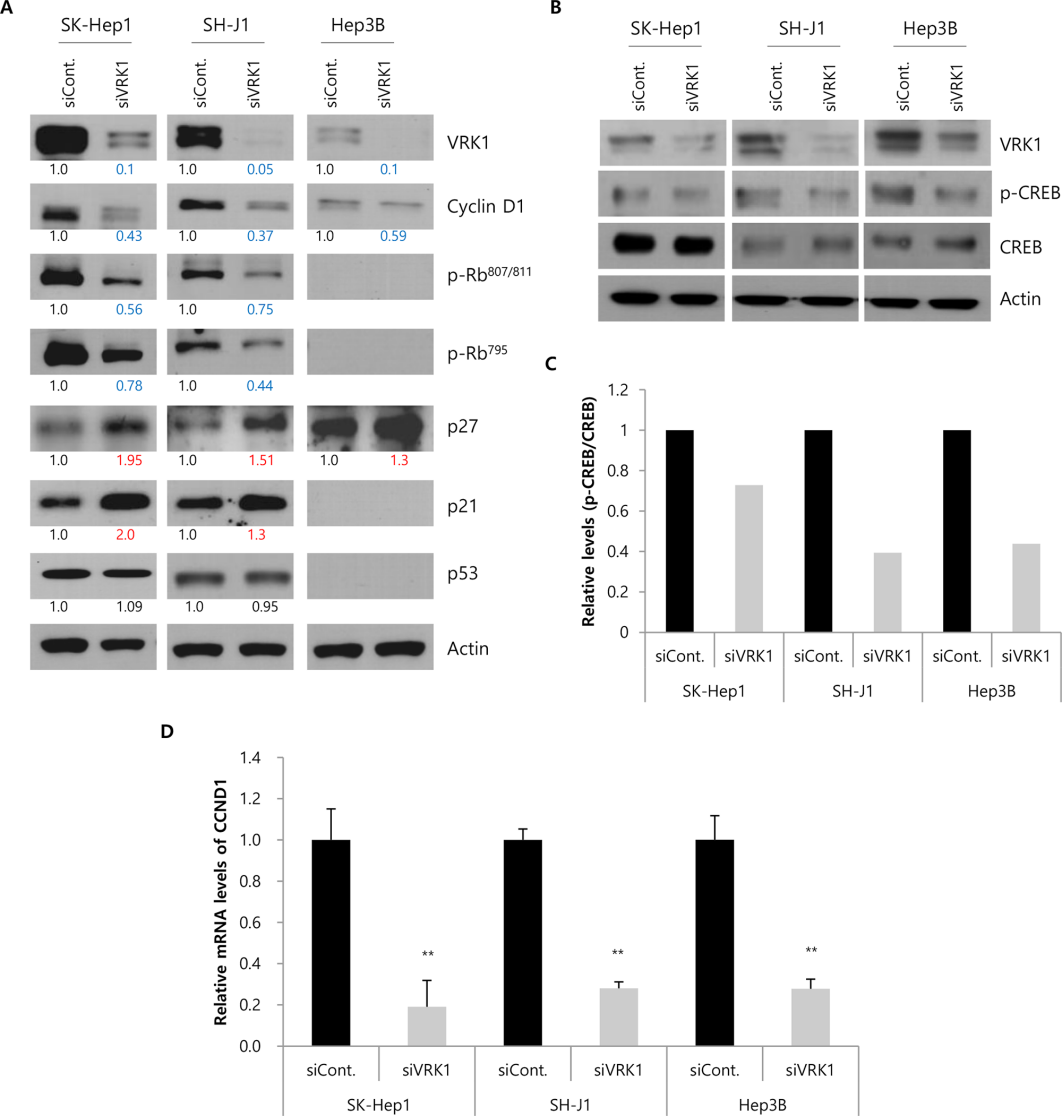
Levels of cell cycle regulators in VRK1-depleted HCC cells **A.** Levels of cell cycle-related proteins and VRK1 were analyzed by Western blotting. Band intensities were quantified by densitometry using Image J and normalized to those of actin serving as the loading control. The intensities were calculated relative to those of the control, which were set to 1.0. Blue and red numbers indicate values lower than 0.8 and higher than 1.2, respectively. **B.** Levels of p-CREB and CREB proteins were confirmed by Western blotting. **C.** Relative levels (p-CREB/CREB) are shown in the histogram. Band intensities were measured by densitometry using Image J, and the intensities of the CREB and p-CREB band were normalized to that of the actin band. **D.** Levels of cyclin D1 mRNA in HCC cells transfected with siRNAs are indicated in the histogram. Cells for Western blotting and RT-PCR were harvested 24 h after transfection. Data shown are representative blots from three independent experiments. (**p < 0.01)

We next assessed the levels of cell cycle regulators associated with G1/S transition in VRK1-depleted cells. Effective silencing of VRK1 led to downregulation of cyclin D1 involved in G1/S transition in HCC cell lines (Fig. 4A). Rb protein is the substrate for cyclin D1 and cdk 4/6 complex, and their phosphorylation is the crucial step for initiating G1/S transition [22]. As we expected, p-Rb^795^ and p-Rb^807/811^ levels were reduced by VRK1 depletion in HCC cells, except in Hep3B cells, which do not express Rb protein (Fig. 4A). Furthermore, levels of p27 and p21, which act as cell cycle inhibitors by suppressing various cyclin/cdk complexes, were increased by VRK1 depletion (Fig. 4A). Because the p21 levels are known to be increased by p53 [23], and p53 is stabilized by VRK1 [10], we checked p53 levels to be increased or decreased in VRK1-depleted HCC cells. However, no significant change of p53 level was observed in VRK1-depleted HCC cells (Fig. 4A). Our group previously showed that cyclin D1 was upregulated by VRK1 through phosphorylation (activation) of CREB, which in turn activates cyclin D1 transcription [8]. We therefore also checked levels of the p-CREB and CREB proteins and cyclin D1 mRNA. We found that p-CREB levels were reduced by VRK1 inactivation in HCC cells (Fig. 4B and 4C), and there was a corresponding reduction the level of cyclin D1 mRNA in VRK1-depleted HCC cells (Fig. 4D).

### Luteolin, a VRK1 inhibitor, reduces HCC growth

**Figure 5:**
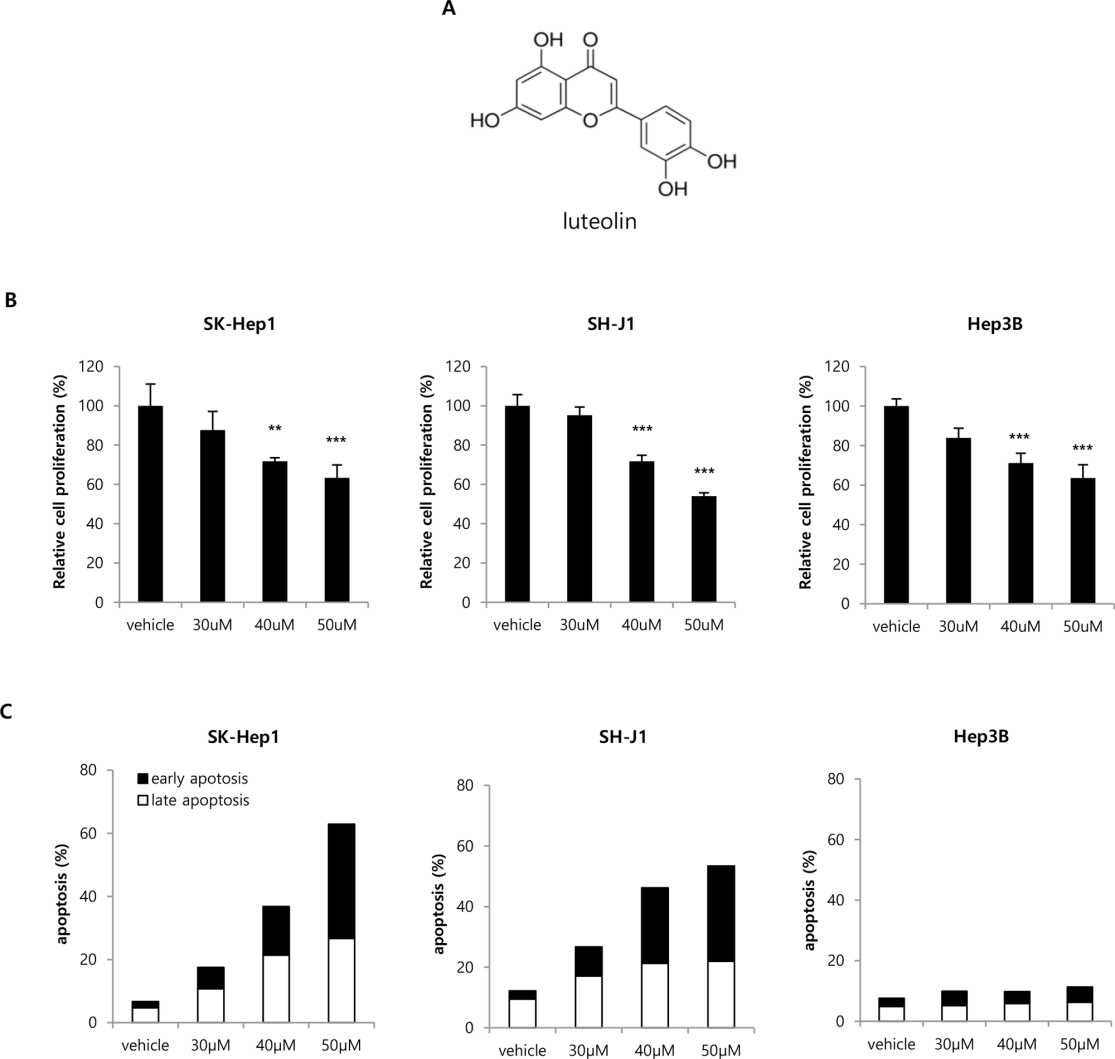
Effect of the VRK1 inhibitor luteolin on HCC cell proliferation and apoptosis **A.** Chemical structure of luteolin. **B.** Concentration-dependent effects of luteolin on cell proliferation were assessed using WST-1 assays after addition of the indicated concentrations of luteolin. SK-Hep1, SH-J1 and Hep3B cells were treated for 24 h with luteolin in the growth medium, after which the culture medium was replaced with one containing WST-1 reagent to measure cell proliferation. HCC cell proliferation was normalized to that of cells treated with vehicle only. **C.** Concentration-dependent effects of luteolin on apoptosis were assessed using the PI and Annexin V double staining method 24 h after addition of luteolin. FITC Annexin V-positive, PI-negative cells were defined as early apoptotic, and FITC Annexin V-positive, PI-positive cells were defined as late apoptotic in the histograms. Raw flow cytometry data are shown in Supplementary Figure 5. Data shown are representative histograms from three independent experiments. (***p < 0.001)

Luteolin is a natural flavonoid (Fig. 5A) originating from plants that has also been shown to inhibit VRK1. Because we found that VRK1 depletion retarded growth of HCC cells, we tested whether a similar retardation could be achieved by pharmacological blockade VRK1. When HCC cells were treated with various concentrations of luteolin, HCC cell proliferation was significantly reduced in a concentration-dependent manner (Fig. 5B). Relative cell proliferation at 40 and 50 μM luteolin was 71.70% ± 1.86 and 63.34% ± 6.58 for SK-Hep1 cells (*P* < 0.01 and *P* < 0.001), 84.71% ± 4.63 and 73.19% ± 3.79 for SH-J1 cells (*P* < 0.001), and 71.18% ± 4.96 and 63.60% ± 6.72 for Hep3B cells (*P* < 0.001; Fig. 5B). Luteolin has also been shown to induce apoptosis in several types of cancers [24]. We therefore tested the ability of luteolin to induce apoptosis in HCC cells. We found that treatment with luteolin significantly and concentration-dependently increased the incidence of apoptosis among SK-Hep1 and SH-J1 cells (Fig. 5C and Sup. 5A). In addition, a minor induction of apoptosis was detected in Hep3B cells treated with luteolin (Fig. 5C and Sup. 5A).

**Figure 6:**
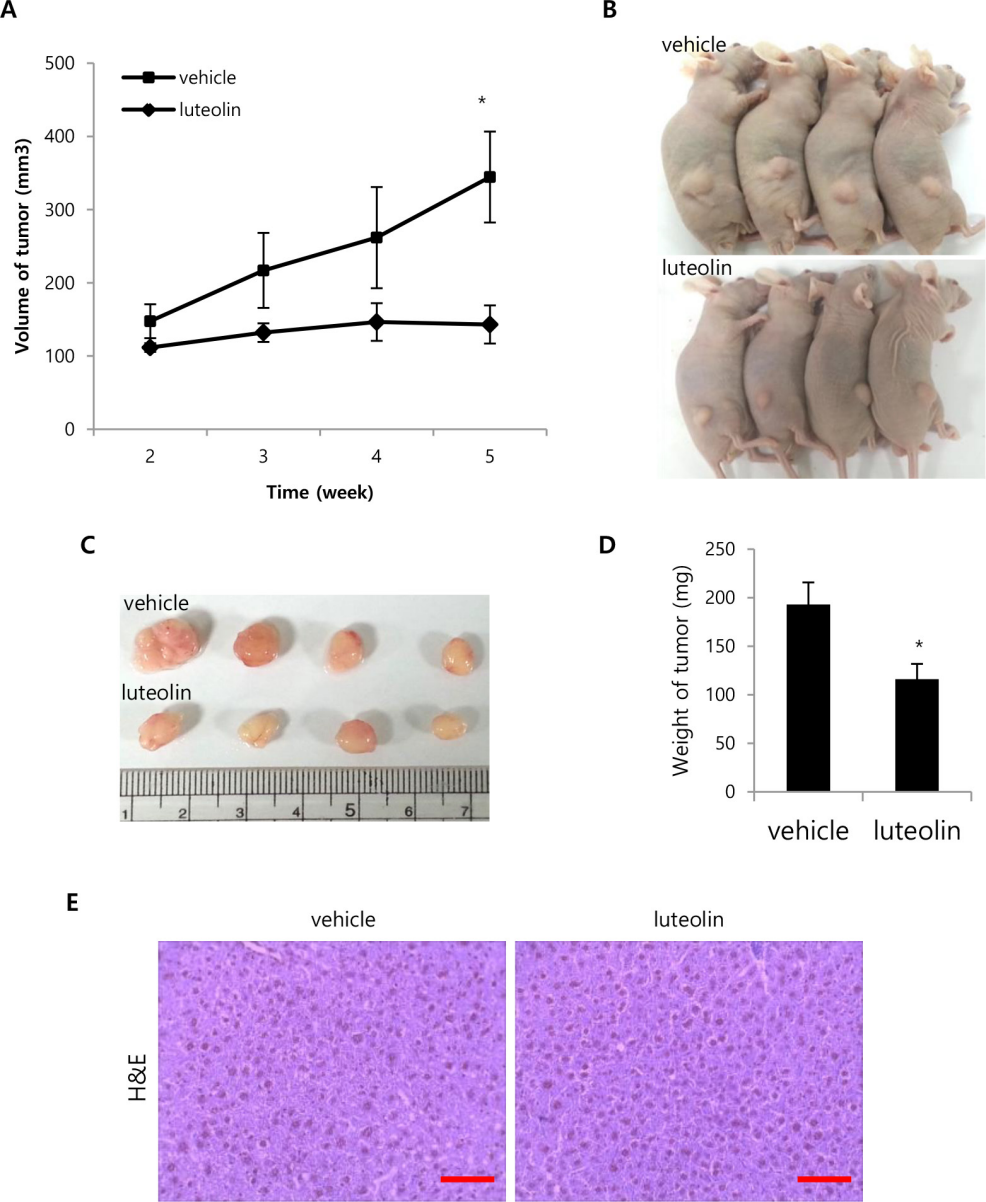
Effect of luteolin on tumor growth *in vivo* **A.** SK-Hep1 cells (1 × 10^7^) were injected subcutaneously. Vehicle or luteolin (20 mg/kg/d) was injected intraperitoneally into mice daily for 2 weeks after inoculation. Tumor size was measured in 4 mice weekly after starting the injections, and are shown as means ± SEM. **B.** Photographs of mice treated with vehicle or luteolin taken after sacrifice. **C.** Tumors were collected from the sacrificed mice, and their sizes were compared. **D.** Tumor weights were measured after sacrifice of mice, and are shown as means ± SEM. **E.** Histological differences in liver tissues from mice treated with vehicle or luteolin were compared using H&E staining. Representative photomicrographs of tumor sections taken at a magnification of 400x. Scale bar indicates 50 μm. (*p < 0.05)

To confirm the anti-tumor effect of VRK1 inhibition *in vivo*, luteolin was administered after subcutaneous injection of SK-Hep1 cells into flanks of nude mice. Tumor volumes were then determined weekly after the injection of luteolin. Significant differences in tumor volume between the luteolin-treated group (143.06 ± 26.03 mm^3^) and the vehicle-treated group (344.47 ± 62.03 mm^3^) were observed 5 weeks after luteolin injection (*P* < 0.05; Fig. 6A). The resulting tumor sizes in mice injected with luteolin were smaller than those injected with vehicle (Fig. 6B and 6C). Correspondingly, tumor weights were smaller in tumors treated with luteolin (116 ± 15.77 mg) than in those treated with vehicle (193 ± 22.80 mg; Fig. 6D). To assess the toxicity of luteolin to mice, samples of liver tissue from vehicle- and luteolin-treated mice were subjected to H&E staining. No histological difference in the liver tissue was observed between the two groups (Fig. 6E).

### High VRK1 expression is associated with a poor prognosis of HCC patients

**Figure 7:**
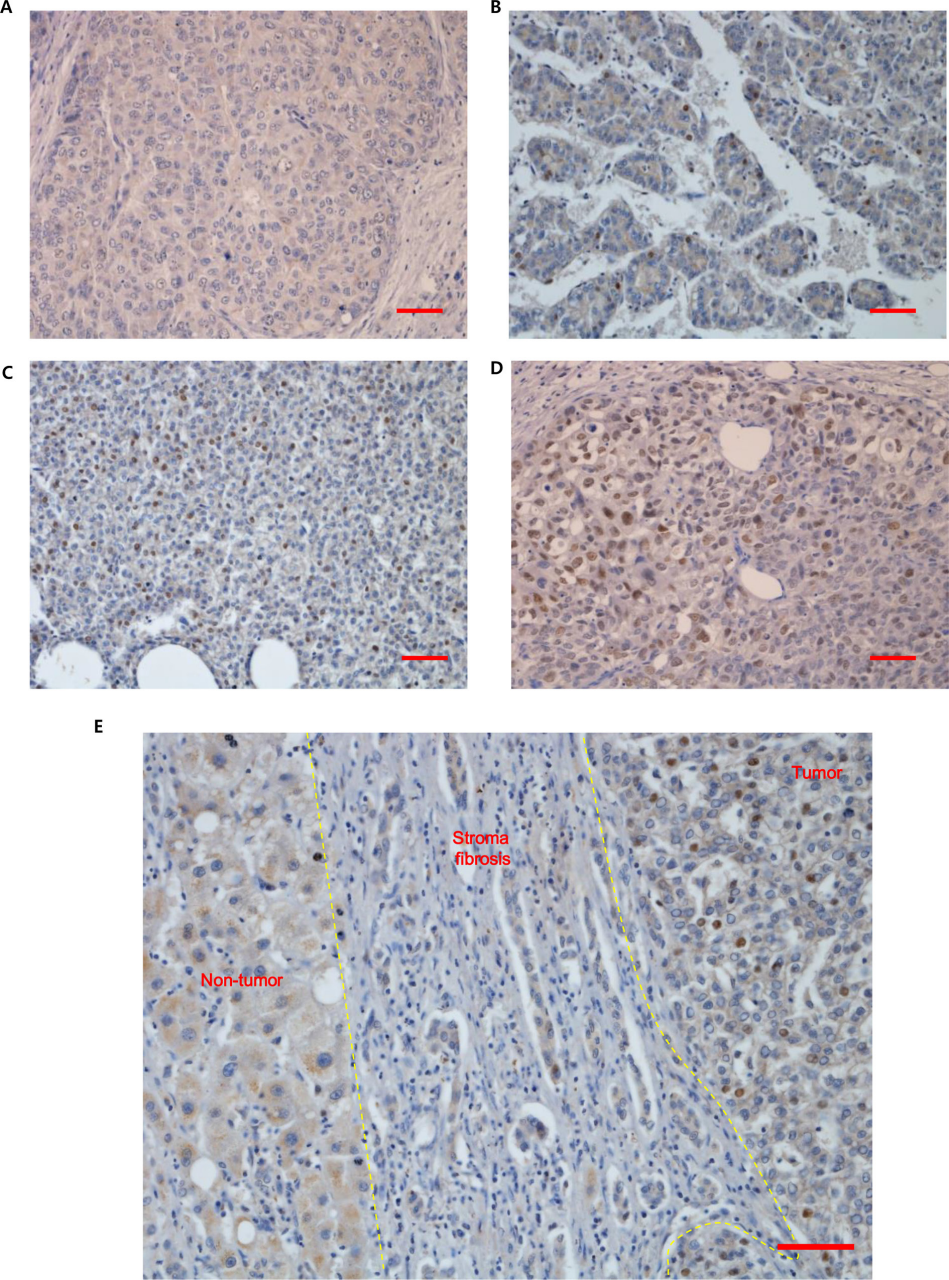
VRK1 expression in non-tumor and HCC tissue **A–D.** VRK1-stained tumor tissues were assigned a score of 0 (A, negative staining), 1 (B, < 5% staining), 2 (C, < 25% staining) or 3 (D, 25–50% staining). **E.** Levels of VRK1 were compared between non-tumor and HCC tissue using immunohistochemistry. Representative images of VRK1-stained tissues were obtained at a magnification of 200x. Scale bar indicates 50 μm.

The fact that VRK1 might be oncogenic in HCC led us to hypothesize that VRK1 could be strongly expressed in HCC patients’ tissues and its expression predictive of a poor prognosis. To test our hypothesis, VRK1 levels were assessed immunohistochemically in 88 HCC specimens. The clinicopathological characteristics of the 88 HCC patients were cataloged (Sup. Table 1), and the intensity of the VRK1 staining in each sample assigned a score of 0 (negative staining, Fig. 7A), 1 (<5% staining, Fig. 7B), 2 (<25% staining, Fig. 7C) or 3 (25–50% staining, Fig. 7D). VRK1 levels were markedly higher in tumors than in non-tumor specimens (0.0000 versus 0.3182; mean 2^−ΔCT^ values, *P* < 0.0001). A representative immunohistochemical image of VRK1 in tumor and non-tumor samples is shown in Fig. 7E. A higher number of VRK1-positive cells was found in the tumor region than the adjacent non-tumor region (Fig. 7E).

**Figure 8:**
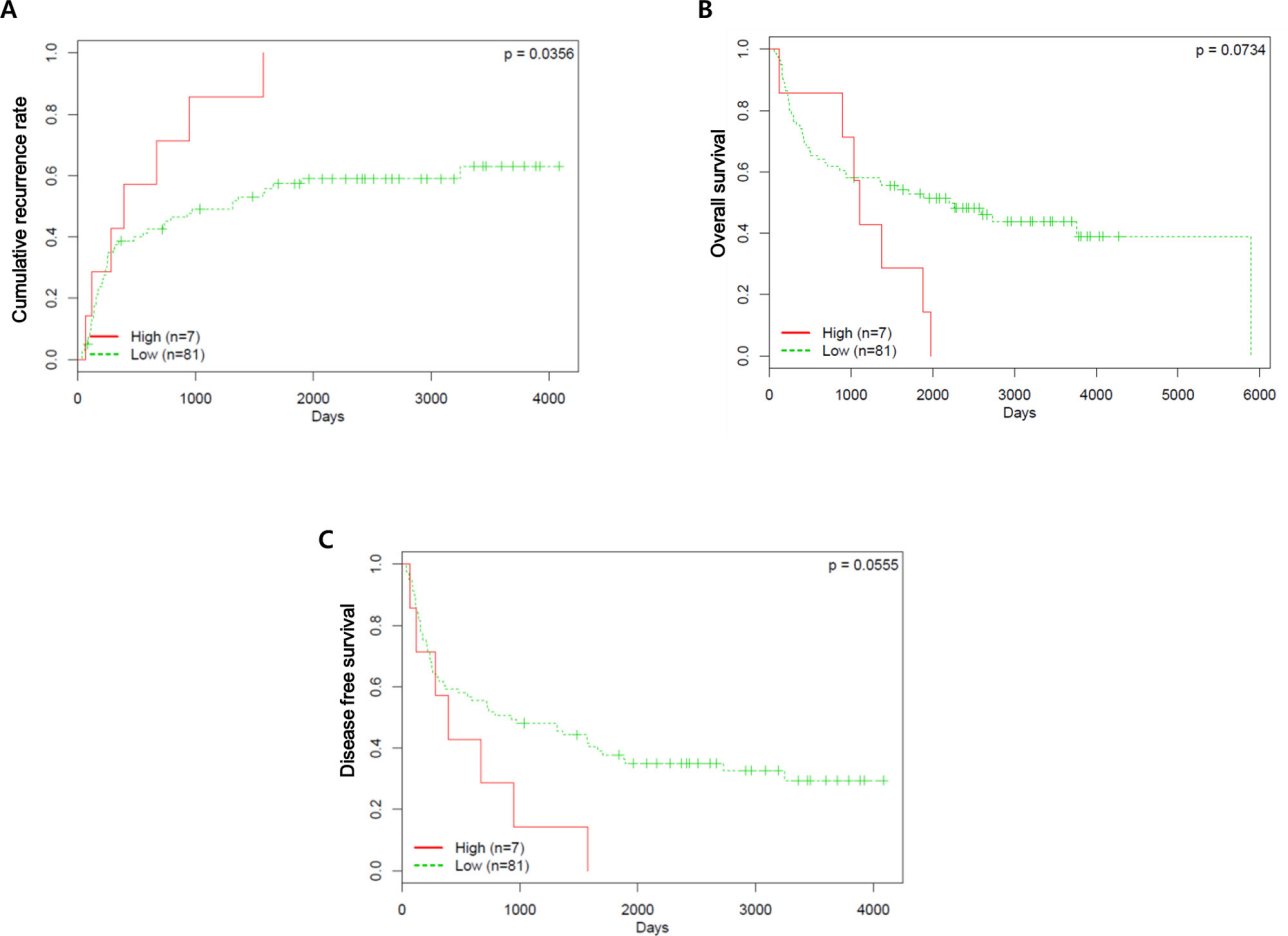
Prognostic value of VRK1 expression in patients with HCC **A–C.** Kaplan-Meier analysis based on VRK1 levels in HCC patients. The probabilities of recurrence (A), overall survival (B) and disease-free survival (C) among patients are shown.

We investigated the association between the VRK1 level and prognosis of HCC patients. Patients were classified as VRK1-high and VRK1-low based on receiver operating characteristic (ROC) curve analysis using the highest area under the curve (AUC) that could significantly discriminate between patients with good and poor prognoses with respect to overall survival (OS). HCCs with IHC intensities of 0–1 and 2–3 were classified as VRK1-low and VRK1-high groups, respectively. Kaplan-Meier survival analysis indicated that median recurrence times in patients with high and low VRK1 levels were 12.85 and 30.64 months, respectively (*P* = 0.0356; Fig. 8A). The VRK1-high group also displayed shorter OS (VRK1-high, 36.43 months; VRK1-low, 53.92 months), but the differences were less significant (*P* = 0.0734; Fig. 8B). The median disease-free survival (DFS) was similar to median recurrence time, with borderline significance (*P* = 0.0555; Fig. 8C). Additionally, we used Fisher's exact test to examine the association between VRK1 levels and clinicopathological characteristics. With the exception of age, there were no significant differences in clinicopathological features, including HBV and HCV status (*P* = 1.0), between the VRK1-high and VRK1-low groups, (Sup. Table 2). The VRK1-high group tended to be ≥ 55 years and VRK1-low group to be < 55 years, with borderline of significance (*P* = 0.051).

The prognostic significance of the VRK1 level was further confirmed in a Cox regression analysis. In a univariate analysis, the AFP level was related to significant risk for both OS and DFS, and liver cirrhosis was the only significant prognostic factor for OS (Table 2). Common prognostic factors for recurrence, OS and DFS were Edmondson grade, tumor size, tumor stage, vascular invasion and tumor number (Table 2). Interestingly, a high VRK1 level was a significant risk factor for recurrence (*P* = 0.041), but not as much for OS and DFS (Table 2). To further confirm VRK1 as an independent prognostic marker for recurrence of HCC, multivariate Cox analysis was performed with the aforementioned significant risk variables for recurrence (Table 3). The multivariate Cox model suggested that VRK1 could be an independent prognostic factor for recurrence with borderline significance (*P* = 0.056; Table 2).

**Table 2:** Univariate Cox analysis

Variable	Univariate analysis Recurrence		Univariate analysis OS		Univariate analysis DFS	
	Hazard Ratio (95% CI)	*P* value	Hazard Ratio (95% CI)	*P* value	Hazard Ratio (95% CI)	*P* value
Age(<55 years vs ≥55 years)	1.06(0.62–1.82)	0.818	1.2(0.69–2.08)	0.526	1.21(0.73–2)	0.466
Gender(male vs female)	1.16(0.66–2.04)	0.615	1.03(0.57–1.85)	0.933	1.13(0.66–1.93)	0.649
Edmondson grade (I,II vs III,IV)	1.94(1.14–3.32)	**0.015**	2.12(1.22–3.69)	**0.008**	1.81(1.09–3)	**0.021**
HBV(absent vs present)	1.1(0.59–2.06)	0.768	1.2(0.63–2.3)	0.579	1.06(0.59–1.91)	0.834
HCV(absent vs present)	0.96(0.38–2.42)	0.935	1.27(0.54–2.99)	0.584	1.25(0.57–2.76)	0.58
AFP level(< 100 ng/ml vs ≥ 100 ng/ml)	1.73(0.97–3.09)	0.063	2.43(1.29–4.6)	**0.006**	1.81(1.05–3.13)	**0.034**
Liver cirrhosis (absent vs present)	0.89(0.51–1.59)	0.703	1.78(1.02–3.13)	**0.043**	1.24(0.74–2.08)	0.412
Tumor size (≤ 5 cm vs > 5 cm)	2.62(1.49–4.61)	**0.001**	2.25(1.27–3.98)	**0.006**	2.1(1.25–3.52)	**0.005**
Tumor stage (I,II vs III,IV)	3.23(1.87–5.57)	**< 0.001**	3.51(1.99–6.18)	**< 0.001**	2.71(1.61–4.58)	**< 0.001**
Vascular Invasion (absent vs present)	3.84(2.01–7.34)	**< 0.001**	4.46(2.21–9.01)	**< 0.001**	3.22(1.8–5.74)	**< 0.001**
Tumor number (Single vs Multi)	2.95(1.71–5.08)	**< 0.001**	3.98(2.26–7.02)	**< 0.001**	3.03(1.81–5.06)	**< 0.001**
VRK1(0,1 vs 2,3)	2.31(1.03–5.15)	**0.041**	2.06(0.92–4.62)	0.08	2.14(0.96–4.75)	0.062

* Data in which *p* < 0.05 are highlighted in bold.

**Table 3:** Multivariate Cox analysis

Variable	Multivariate analysis Recurrence	
	Hazard Ratio(95% CI)	*P* value
Edmondson grade (I,II vs III,IV)	1 (0.55–1.81)	0.999
Tumor size (≤5 cm vs >5 cm)	1.7 (0.9–3.19)	0.1
Tumor stage (I,II vs III,IV)	1.04 (0.34–3.22)	0.946
Vascular Invasion (absent vs present)	2.77	**0.009**
Tumor number (Single vs Multi)	2.29 (0.81–6.45)	0.118
VRK1 (0,1 vs 2,3)	2.37 (0.98–5.76)	**0.056**

* Data in which *p* < 0.05 are highlighted in bold.

## DISCUSSION

The present study was conducted to elucidate the role played by VRK1 in HCC cell lines and patient tissues, and the results highlight the oncogenic role of VRK1 in HCC. VRK1 regulates the cell cycle by controlling levels of effector proteins associated with G1/S transition. VRK1 levels, which were higher in HCC than healthy tissue, were associated with shorter OS and DFS and a higher recurrence rate in HCC patients.

Depletion of VRK1 using siRNA and shRNA suppressed the growth of HCC cells significantly. Consistent with that finding, VRK1 depletion was previously shown to suppress cell proliferation in WS1 fibroblastic cells and lung cancer cells [25, 26], and to reduce tumor size in breast cancer *in vivo* [17]. We observed the most significant effect of VRK1 knockdown on HCC cell growth in SK-Hep1 cells, which expressed the highest level of VRK1. Conversely, transient overexpression of VRK1 did not significantly affect SK-Hep1 cell proliferation. The smallest effect of VRK1 knockdown was on Hep3B cells, which expressed lowest levels of VRK1. Similarly, growth of THLE-2 cells, a normal liver cell line expressing low levels of VRK1, was also not significantly affected by VRK knockdown. Thus the differential sensitivities of cells to VRK1 knockdown or overexpression appears to reflect differences in basal VRK1 levels.

Xenograft assays confirmed that depletion of VRK1 could suppress tumor growth *in vivo*. Both VRK1 and Ki-67 have been identified within proliferating areas of squamous epithelium, and high levels of VRK1 correlated positively with Ki-67 in head and neck squamous carcinomas [15]. Similarly, we showed here that VRK1-positive cells within tumors in mice co-located with Ki-67-positive cells. Furthermore, the number of Ki-67 positive cells was significantly lower in tumors derived from VRK1-depleted SK-Hep1 cells than tumors derived from intact SK-Hep1 cells. Taken together, these findings suggest that VRK1 expression affects the proliferation of tumor cells, that VRK1 knockdown suppresses HCC cell growth *in vitro* and *in vivo*, and that the extent of its effect differs depending upon the basal VRK1 level in HCC cells.

We found that siRNA-mediated depletion of VRK1 caused G1 arrest in HCC cells, which is consistent with previous observations in H460 and H1299 lung cancer cells and MCF 10A mammary epithelial cells [17, 26]. VRK1 also had distinct functions during G2/M phase, including phosphorylation of proteins involved in regulating the nuclear envelope, chromatin condensation and Golgi fragmentation [3]. However, the number of cells in G2/M phase was unaffected by VRK1 depletion in an asynchronous cell population (Fig. 3A). This suggests that during cell cycle progression in HCC cells, VRK1 function during G1/S phase may be more important than during G2/M phase, or other mitotic kinases such as the Aurora kinases and haspin may compensate for the loss of VRK1. Indeed, overexpression of Aurora A and B has been reported in HCC [27, 28]. Among SK-Hep1 and SH-J1 cells, which normally express high levels of VRK1, VRK1 depletion with nocodazole treatment significantly increased numbers of G1-arrested cells, but this effect was not as significant in Hep3B cells, which express very low levels of VRK1 (Fig. 3C and 3D). Interestingly, the percentage of G1 gated cells gradually declined in VRK1-depleted Hep3B cells (Fig. 3C and 3D), which implies VRK1 depletion led to delayed G1/S transition in Hep3B cells. These findings may be explained by the different genetic backgrounds of the three cell lines. As in an earlier study of Rb-null Hep3B cells [29], we failed to detect Rb proteins in Hep3B cell lysates (Fig. 4A). Because the accumulation of p-Rb by cyclin D and Cdk complexes could be a critical regulatory step for G1/S transition [22], the reduction in cyclin D1 levels may not be as significant for G1/S phase transition in Rb-null cells as in Hep3B cells. Thus, the extent of its effect may be differently manifest, depending on the genetic background of the HCC cells. Levels of cyclin D1, p-Rb^795^ and p-Rb^807/811^ were all reduced in VRK1-depleted cells, which is consistent with an earlier report showing that cyclin D1 and p-Rb levels declined upon VRK1 downregulation in fibroblasts [25]. The reduction in the levels of cyclin D1 was attributed to a decrease in its gene's transcription, which was in turn related to decreased CREB phosphorylation in VRK1-depleted HCC cells. We also observed that both p21 and p27 were upregulated upon VRK1 knockdown, but that p53 levels were unchanged (Fig. 4A). VRK1 thus appears to regulate p21 expression independently of p53, though the mechanism by which VRK1 affects expression of p21 and p27 remains to be clarified. Taken together, these findings indicate that VRK1 depletion causes arrest or delay of G1/S transition, leading to a significant increase in the HCC cell fraction in G1 phase. This suggests the retardation of HCC cell growth induced by VRK1 depletion reflects interference with the cell cycle.

We suggest that VRK1 is a potential therapeutic target for treatment of HCC, based on its levels in our clinical samples and its role in cell proliferation. It has also been proposed that in lung cancer and rhabdomyosarcoma, the VRK1 gene is a “druggable target gene” whose function in tumors is distinguishable from that in healthy tissue [26, 30]. In the present study, the VRK1 inhibitor luteolin effectively inhibited HCC cell proliferation. Although several kinase inhibitors have been shown to suppress VRK1 kinase activity, the inhibitory mechanism has not been determined [31]. Our group recently reported that luteolin specifically binds to the catalytic domain of VRK1 to inhibit the kinase activity [32]. By inhibiting VRK1, luteolin reduced HCC cell growth *in vitro* and *in vivo*, and also induced apoptosis in HCC cells (Fig. 5C). Consistent with our results, the anti-cancer effects of luteolin have also been demonstrated in several HCC cell lines. For example, luteolin inhibited HGF-induced HepG2 cell invasion and mediated increases in intracellular ROS levels in Huh-7 cells [33, 34]. In the present study, however, we did not observe significant induction of apoptosis upon VRK1 depletion by VRK1 siRNA (Sup. Fig. 4). The extent of its different effect on apoptotic induction between VRK1 inhibition with luteolin treatment and VRK1 depletion by VRK1 siRNA could be explained by multiple molecular target of luteolin [24] or/and the residual VRK1 expression in cells transfected with VRK1 siRNAs. Addressing this issue will require development of more specific and potent inhibitors of VRK1.

Aberrant VRK1 expression has been reported in colon cancer and lung cancer tissues [26, 35]. Similarly, we observed higher levels of VRK1 in HCC tumor tissue and cell lines than in corresponding non-tumorous liver tissue and normal liver cells (Fig. 1A and Fig. 7E). VRK1 expression is known to be dependent on the p53 status. Induction of p53 expression using UV light or transfection with the plasmid encoding p53 downregulated VRK1 levels, which is consistent with the inverse correlation between VRK1 and p53 levels reported previously [12]. Moreover, VRK1 accumulated in lung tumors expressing mutant p53 with an altered regulatory loop [16]. In contrast to those earlier reports, we did not find an inverse correlation between VRK1 and p53 levels in HCC cell lines (Fig. 1A and Table 1); nonetheless, the regulation of VRK1 expression by p53 was apparent. For example, VRK1 levels were reduced by ectopic expression of p53 in HCC cells (Sup. Fig. 1), and VRK1 increased the stability of p53 by phosphorylating it on Thr 18, which inhibits p53 binding to mdm2 [10, 36]. On the other hand, we detected no significant change in p53 levels in response to VRK1 depletion in HCC cells (Fig. 4A). Under our experimental conditions, the cells were not physiologically stressed, which may make it more difficult to detect changes in p53 levels related to VRK1 depletion.

An association between stronger VRK1 expression with shorter OS was previously observed in patients with estrogen receptor-positive breast cancer [20]. Kaplan-Meier survival analysis showed that HCC patients expressing high levels of VRK1 likely experience shorter times of OS and DFS and a higher incidence of recurrence. A high VRK1 level was found to be a significant risk factor for recurrence, as were other common prognostic factors, including Edmonson grade, tumor size and tumor stage. In addition, moderate hazard ratios for OS and DFS were also found. It is noteworthy that the multivariate Cox analysis suggested that the VRK1 level could be an independent prognostic factor for recurrence with borderline significance (*P* = 0.056).

In sum, we found that VRK1 is up-regulated in HCC, and its increased expression is associated with poor prognosis. *In vitro* and *in vivo* studies indicate that depletion of VRK1 leads to G1 arrest and inhibition of HCC cell proliferation. In addition, VRK1 depletion may suppress cyclin D1 expression by downregulating CCND1 transcription and activating p21 and p27 expression. Notably, luteolin, a VRK1 inhibitor, suppresses HCC tumor growth by inhibiting cell proliferation and inducing cell death. Taken together, these findings suggest that high VRK1 levels could potentially serve as an indicator of poor prognosis and/or a therapeutic target in HCC.

## MATERIALS AND METHODS

### Cell culture and WST-1 assay

The SK-Hep1, Hep3B, Huh-7, HepG2 and SNU449 cell lines were obtained from the Korean Cell Line Bank (Korea). SH-J1 cells were provided by Dr. Dae-Ghon Kim (Medical School, Chonbuk National University) [37]. HCC cells were cultured at 37°C under 5% CO_2_ in DMEM supplemented with 10% FBS, 100 units/ml of penicillin and 100 μg/ml streptomycin. THLE-2 cell line was purchased from the American Type Culture Collection (USA). THLE-2 cells originated from human primary normal liver cells were plated on culture plates pre-coated with a solution containing 0.01 mg/mL fibronectin, 0.03 mg/mL bovine collagen type I and 0.01 mg/mL bovine serum albumin dissolved in Bronchial Epithelium Basal Medium (BEBM, Lonza). THLE-2 cells were cultured at 37°C under 5% CO_2_ in BEBM supplemented with BEGM SingleQuots (Lonza).

The effects of VRK1 overexpression or depletion on proliferation were measured using WST-1 assays. Culture medium was exchanged with medium containing WST-1 reagent (Roche, 1:10 final dilution) at 0 and 72 h after transfection. After incubating cells for 90 min at 37°C, absorbance at 450 nm was measured using a microplate reader (Spectrafluor Plus, Tecan). The effect of luteolin on HCC cells was also assessed using WST-1 assays. Cells were plated and incubated in medium containing various concentrations of luteolin (30, 40 and 50 μM). After 24 h of treatment, the medium was replaced with medium containing WST-1 reagent, and the cells were incubated for 1 h at 37°C.

### Colony formation assay

Cells were transfected with the indicated siRNAs. After 24 h of transfection, the cells were seeded at a density of 2 × 10^3^ cells per well in 6-well dishes and transfected again 5 days later. Ten days after the initial transfection, cells were fixed for 5 min with 4% formaldehyde in PBS, washed with PBS, and stained for 1 h with 0.5% crystal violet in 25% methanol. Plates were then washed with PBS to remove excessive dye and photographed with a digital camera. Quantitative changes in clonogenicity were determined by extracting colonies with 20% acetic acid and measuring the absorbance at 595 nm. For stable cell lines, 2 × 10^3^ cells were plated, and cells were fixed and stained 10 days after plating.

### Expression vectors and synthesis of siRNA

The VRK1 and p53 expression vectors were used as described previously [4, 38]. Small interfering RNA (siRNA) duplex targeting human VRK1 (siVRK1; catalog no. 16704) and control scrambled siRNA (siCont.; catalog no. SN-1003) were purchased from Ambion (USA) and Bioneer (Korea), respectively.

### Real-time RT-PCR

Real-time RT-PCR was performed to measure the cyclin D1 mRNA levels in HCC cells as described previously [39]. Briefly, total RNA was isolated from the HCC cells using a RNeasy mini kit (Qiagen) according to the manufacturer's instructions, and reversely transcribed to cDNA using MMLV reverse transcriptase and the oligo(dT) primer kit (Solgent). Actin was used as the control gene. Real-time PCR was performed using SYBR Green I (Takara) and a Light-Cycler rapid thermal cycler (Roche Diagnostics). The primer sequences are shown in Supplementary Table 3. The relative VRK1 levels in HCC cells were determined using the 2^−ΔΔC^_T_ method, as described [40].

### Western Blot analysis

Western blot analysis was performed as described previously [41] using following antibodies: mouse anti-VRK1 (Santa Cruz, sc-271062), goat anti-actin (Santa Cruz, sc-1615), rabbit anti-cyclin D1 (Santa Cruz, sc-753), rabbit anti-cyclin D2 (Santa Cruz, sc-181), rabbit anti-CREB (Cell signaling, 9198), rabbit anti-p-CREB (Cell signaling, 9191), rabbit anti-p27 (Santa Cruz, sc-528), mouse anti-p21 (Santa Cruz, sc-6246), rabbit anti-p-Rb^S795^(Cell signaling, 9301), mouse anti-p53 (Santa Cruz, sc-126) and rabbit anti-p-Rb^S807/S811^(Cell signaling, 9308).

### Cell cycle analysis

To analyze the progression of the cell cycle on asynchronous cell populations, 8 × 10^5^ cells were seeded onto 6-well plates, transiently transfected with negative control or VRK1-specific siRNA and grown for 24 h. Cells were then collected by trypsinization, fixed in the fixation solution containing 70% ethanol and 0.5% Tween-20, washed in PBS containing 1% BSA, resuspended in 200 μl PBS containing 100 μg/ml RNase (Sigma) and 50 μg/mL propidium iodide (Sigma), incubated in the dark for 40 min at room temperature, and analyzed using the Canto II flow cytometer (BD Biosciences). Acquired data were analyzed using ModFit LT software (Verity Software House).

To synchronize the cells at G2/M phase, cells were exposed to nocodazole (50 ng/ml) at 24 h after transfection. After the indicated exposure times (Fig. 3C), flow cytometry were performed using the same procedure described above.

### Cell death analysis

Apoptosis was measured using a FITC Annexin V Apoptosis Detection Kit (BD Biosciences, 556547). HCC cells were treated with selected concentrations of luteolin or transfected with the indicated siRNAs and stained with propodium iodide (PI) and Annexin-V FITC. The stained cells were analyzed using the Canto II flow cytometer (BD Biosciences).

### Reagents

Lipofectamine 2000 (Invitrogen) was used to introduce expression vectors and siRNAs into cells. Luteolin and nocodazole were purchased from Santa Cruz and Sigma Aldrich, respectively.

### Establishment of stable cell lines

Stable cell lines were established by transduction with lentiviral shRNA targeting VRK1 or scrambled control (Sigma). SK-Hep1 cells (1 × 10^5^) were seeded onto 6-well plates and infected with lentiviral particles containing scrambled control shRNA or VRK1 shRNA clones 1 to 5 at a multiplicity of infection (MOI) of 2 in DMEM containing 8 μg/ml polybrene (Sigma) in each well. After 1 day, the DMEM containing lentivirus was replaced with growth medium, and the cells were incubated for an additional 1 day. Puromycin (2 μg/ml; Invitrogen) was added to each well to select for cells expressing shRNA. Knockdown efficiency in each stable cell line was confirmed by Western blot analysis performed 2, 3, 4, 5 and 6 weeks after viral transduction (Fig. 2A and Sup. Fig. 2). The target sequences of the five shRNA clones are shown in Supplementary Table 4.

### Xenograft assay

Five-week-old male nude mice were purchased from Orient Bio (Korea) and raised in a specific pathogen-free area within the animal facilities at POSTECH (Korea). All animals were handled according to Institutional Animal Care and Use guidelines of POSTECH. To compare tumor growth between established stable SK-Hep1 cell lines *in vivo*, 2 weeks after viral transduction, the established cells (5 × 10^6^) were resuspended in serum-free DMEM with Matrigel basement membrane matrix (BD Biosciences) at a 1:1 ratio (total volume, 100 μl) and subcutaneously injected into the right and left flanks of nude mice. Tumor sizes were measured every 2 weeks using the Vernier caliper, and volumes were determined according to the formula L × S^2^ × 0.52, where L is the longest diameter and S the shortest diameter of the tumor. Mice were sacrificed 8 weeks after injection and the solid tumors isolated.

The effect of luteolin on tumor growth *in vivo* was assessed using the method described previously [42] with minor modification. SK-Hep1 cells (1 × 10^7^) were resuspended in serum-free DMEM with Matrigel basement membrane matrix (BD Biosciences) at a 1:1 ratio (total volume, 100 μl) and subcutaneously injected into the right flank of nude mice. Mice were then intraperitoneally administered vehicle or luteolin (20 mg/kg/d) daily from 2 weeks after inoculation. The body weight and tumor size of each mouse were measured and tumor volumes were determined according to the formula L × S^2^ × 0.52, where L is the longest diameter and S the shortest diameter of the tumor.

### Patients and tissue samples

HCC and surrounding non-tumor hepatic tissues were collected with informed consent from 88 HCC patients who had undergone curative resection of primary HCCs between 1995 and 2007 at the Ajou Medical Center in Korea. The clinicopathological data for the 88 patients, which are summarized in Supplementary Table 1, were almost entirely available in the medical records. Exceptions were the HBV status in one case, HCV status in two cases and AFP level in one case. BCLC stage, tumor stage, and Edmondson and Steiner grades were used according to published criteria. Liver function was preserved in all patients, and no patients died within 2 months after surgery in the current cohort. The median follow-up time was 23.47 months (range, 1.15–134.30) for recurrence and DFS and 47.77 months (range, 2.01–193.68) for OS. Recurrence was defined as the first appearance of new lesions at any site after surgery, as confirmed using radiologic imaging. Patients who underwent local therapy, including transcatheter arterial chemoembolization and radiofrequency ablation, were not included. Fresh tumors and surrounding normal tissues were partly snap-frozen in liquid nitrogen immediately after hepatectomy and stored at −80°C. The study protocol was approved by the Institutional Review Board of the Ajou Medical Center.

### Immunohistochemistry

Immunohistochemical detection of VRK1 in patient samples was performed using formalin-fixed and paraffin-embedded tissue sections (∼4 μm thick). After deparaffinization in xylene for 15 min, rehydrated tissue sections were subjected to antigen retrieval by boiling in Tris-EDTA buffer (pH 9.0) for 5 min. Slides were then incubated with rabbit anti-VRK1 (Santa Cruz) for 1 h at room temperature, and the VRK1 antigen-antibody reaction was detected using a Real Detection System (Dako, K5001). Immunohistochemical intensity of VRK1 staining was evaluated by two independent pathologists. VRK1 expression was evaluated in 10 high-power fields (400 ×). In each sample, intensity was classified as 0 (negative staining), 1 (<5% staining), 2 (<25% staining), 3 (25–50% staining) and 4 (>50% staining), and the average intensity assessed. Immunohistochemical detection of Ki-67 in mouse tumors treated with luteolin was also performed using the same method described above with mouse anti-Ki-67 (Dako, M7240) antibody.

### Immunofluorescence

Solid tumors from mice were placed in formalin for paraffin block preparation. To verify Ki-67-positive and VRK1-positive cells in the same tumor section, solid tumor sections were incubated with mouse anti-Ki-67 (Dako, M7240) and rabbit anti-VRK1 antibodies prepared as described [43]. Sections were subsequently washed with PBS containing 0.05% Tween-20 and incubated with anti-rabbit Alexa Fluor 594 (Invitrogen) and anti-mouse Alexa Fluor 488 (Invitrogen) for 1 h at room temperature in the dark. Sections were then washed with PBS containing 0.05% Tween-20, incubated with Hoechst 33342 (Invitrogen) for 2 min at room temperature and mounted with Mounting medium (Dako).

### Hematoxylin and Eosin (H&E) Staining

Liver and tumor tissues from mice were fixed and embedded in paraffin and ∼4-μm-thick were prepared. The sections were stained with H&E as described previously [44].

### Statistical analysis

Relations between VRK1 protein levels and clinicopathological characteristics were evaluated using χ^2^ and Fisher's exact tests. Tumor recurrence, OS and DFS were compared between VRK1-high and VRK1-low groups using the Kaplan–Meier method, and significant differences in curves was assessed using the log-rank test. Results of the WST-1 and colony forming assays, weight and volume of tumors, immunofluorescence intensities and real time RT-PCR were analyzed using Student's *t* test. Cox regression analysis was performed to evaluate the prognostic significance of clinicopathological parameters and VRK1 expression. Values of *P* < 0.05 were considered statistically significant, and data marked with a one (*), two (**) or three (***) asterisks indicate *P* values of < 0.05, < 0.01 and < 0.001, respectively. Statistical analyses were performed using SPSS v. 18.0 (IBM) and the open source statistical program R v 3.1.1.

## SUPPLEMENTARY FIGURES AND TABLES


