# Supplementary material for: Vaccinia-related kinase 1 promotes hepatocellular carcinoma by controlling the levels of cell cycle regulators associated with G1/S transition
Source: Oncotarget. 2015 Sep 7;6(30):30130–48. doi: 10.18632/oncotarget.4967 (PMC4745786; doi:10.18632/oncotarget.4967)
Supplement: Supplementary file 1 [file oncotarget-06-30130-s001.pdf]

SUPPLEMENTARY FIGURES AND TABLES

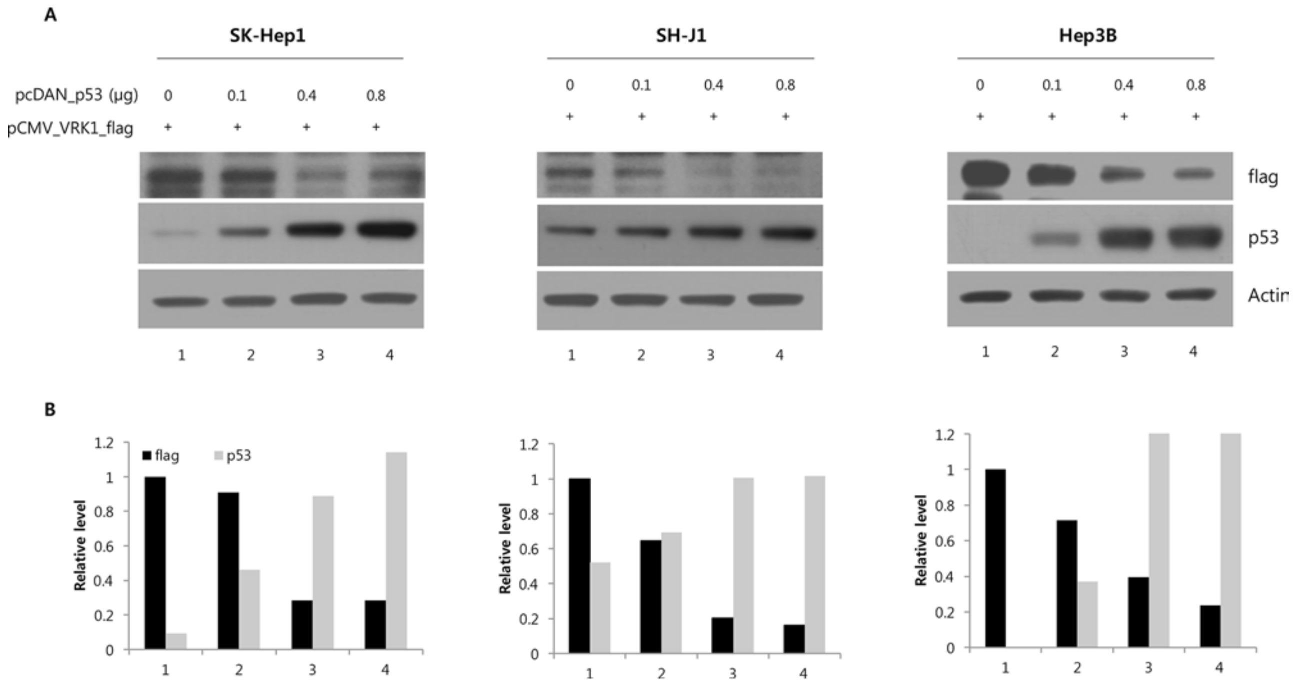

Supplementary Figure S1: Dose-dependent effect of p53 on VRK1 levels in HCC cell lines. A. SK-Hep1, SH-J1 and Hep3B cells were transfected with increasing amounts of pcDNA_p53, as indicated, and 0.2 μg of pCMV_VRK1-flag. Levels of VRK1-flag and p53 in SK-Hep1, SH-J1 and Hep3B were analyzed using Western blotting 36 h after transfection. **B.** Band intensities were quantified by densitometry using Image J and normalized to intensity of the actin band used as a loading control. The band intensities were calculated relative to the VRK1 band in lane 1, which were set to 1.0.

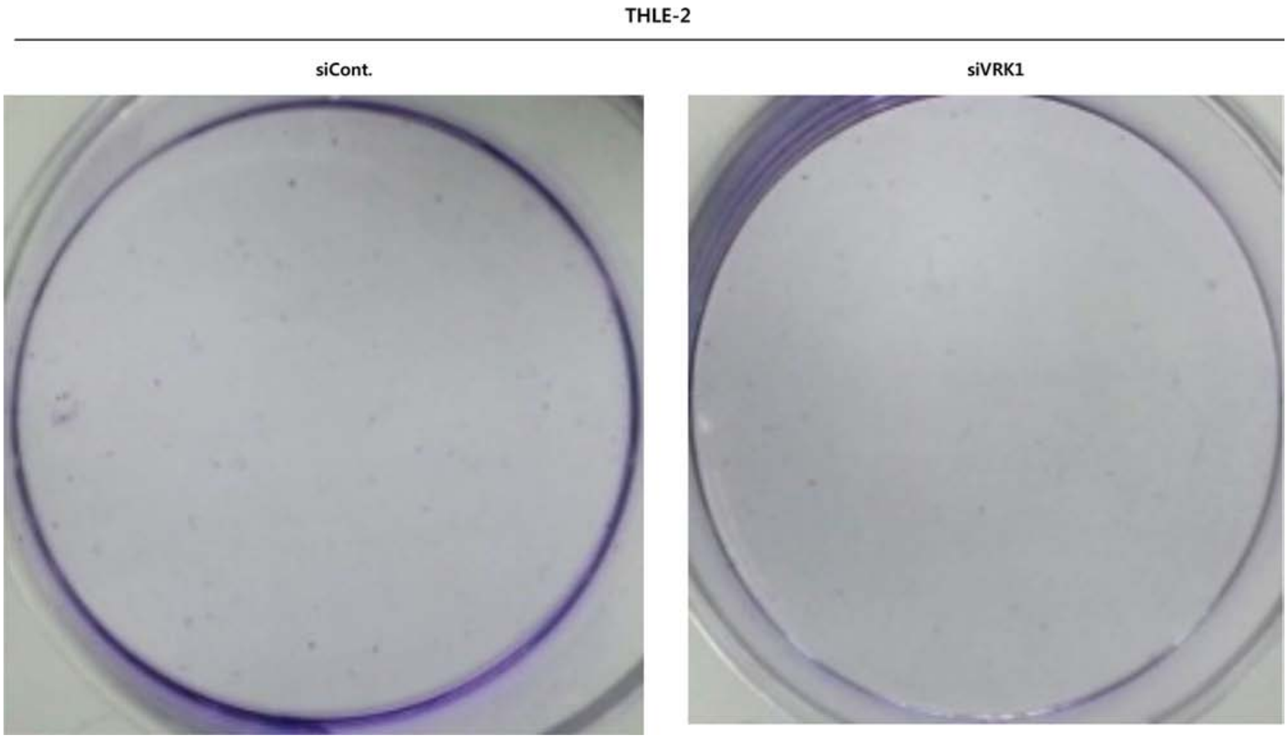

Supplementary Figure S2: Representative image showing colony formation by THLE-2 cells transfected with siCont or siVRK1.

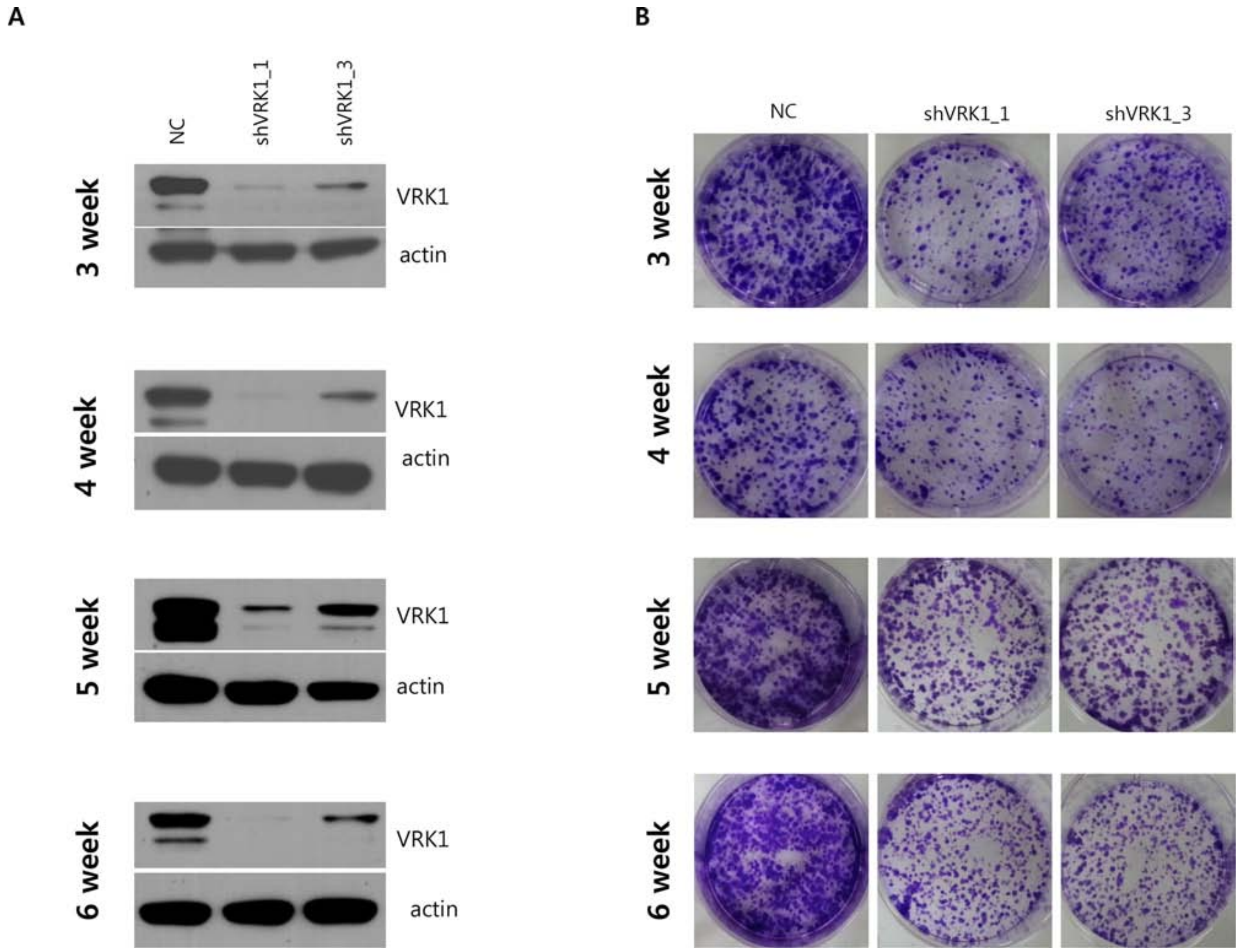

Supplementary Figure S3: Growth of VRK1-depleted SK-Hep1 cells. A. VRK1 depletion efficiencies were compared by Western blotting 3, 4, 5 and 6 weeks after lentiviral transduction **B.** Efficient lentiviral particles (Clones 1 and 3) targeted to different sequences of the VRK1 gene were used for colony formation assays 3, 4, 5 and 6 weeks after transduction.

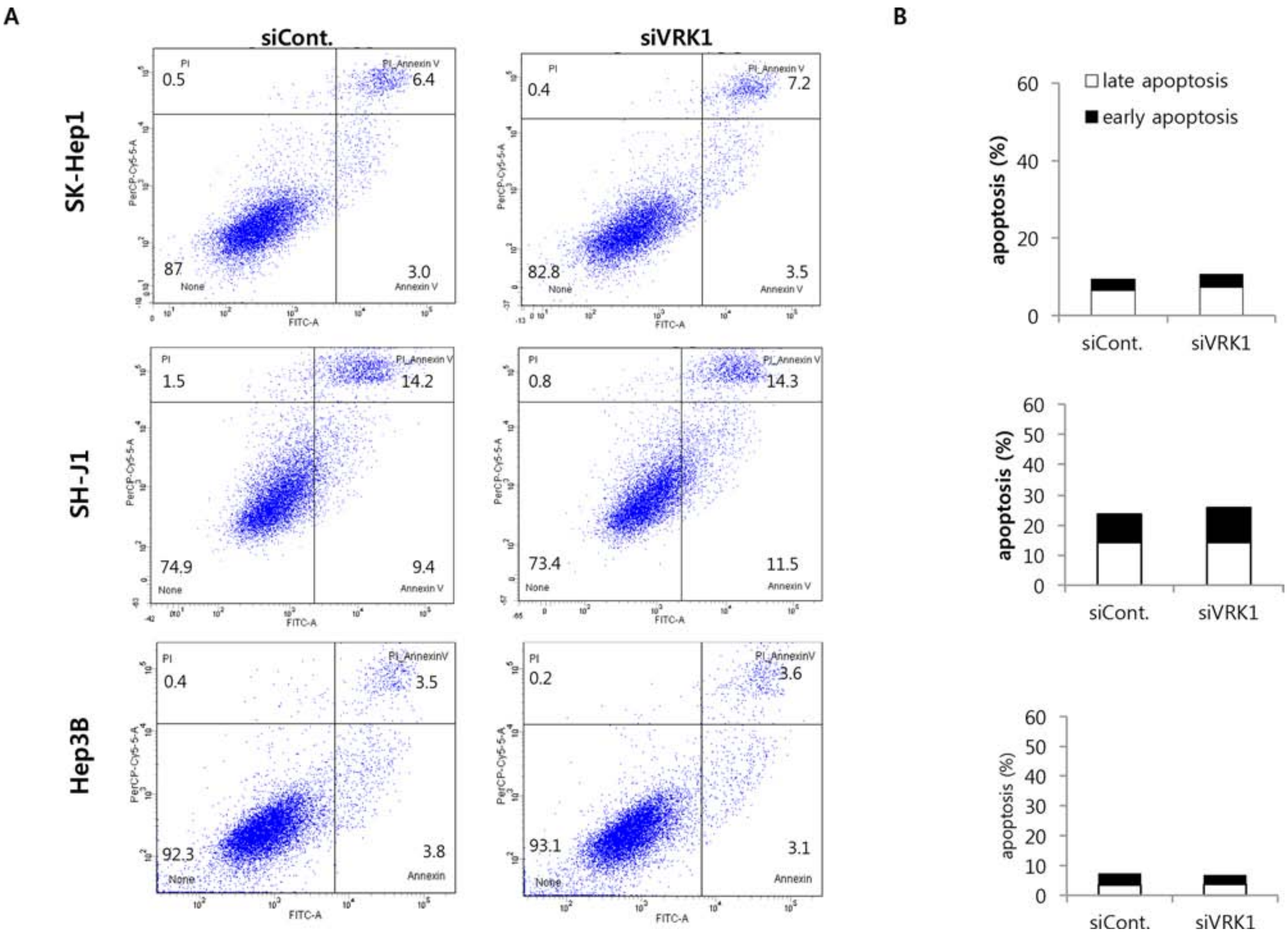

Supplementary Figure S4: Effect of VRK1 depletion on HCC cell apoptosis. A. Effects of VRK1 depletion on apoptosis were assessed using PI and Annexin V double staining followed by flow cytometry 24 h after transfection. **B.** Annexin V-positive, PI-negative cells were defined as early apoptotic, and Annexin V-positive, PI-positive cells were defined as late apoptotic in the histograms. Data shown are representative histograms from three independent experiments.

A

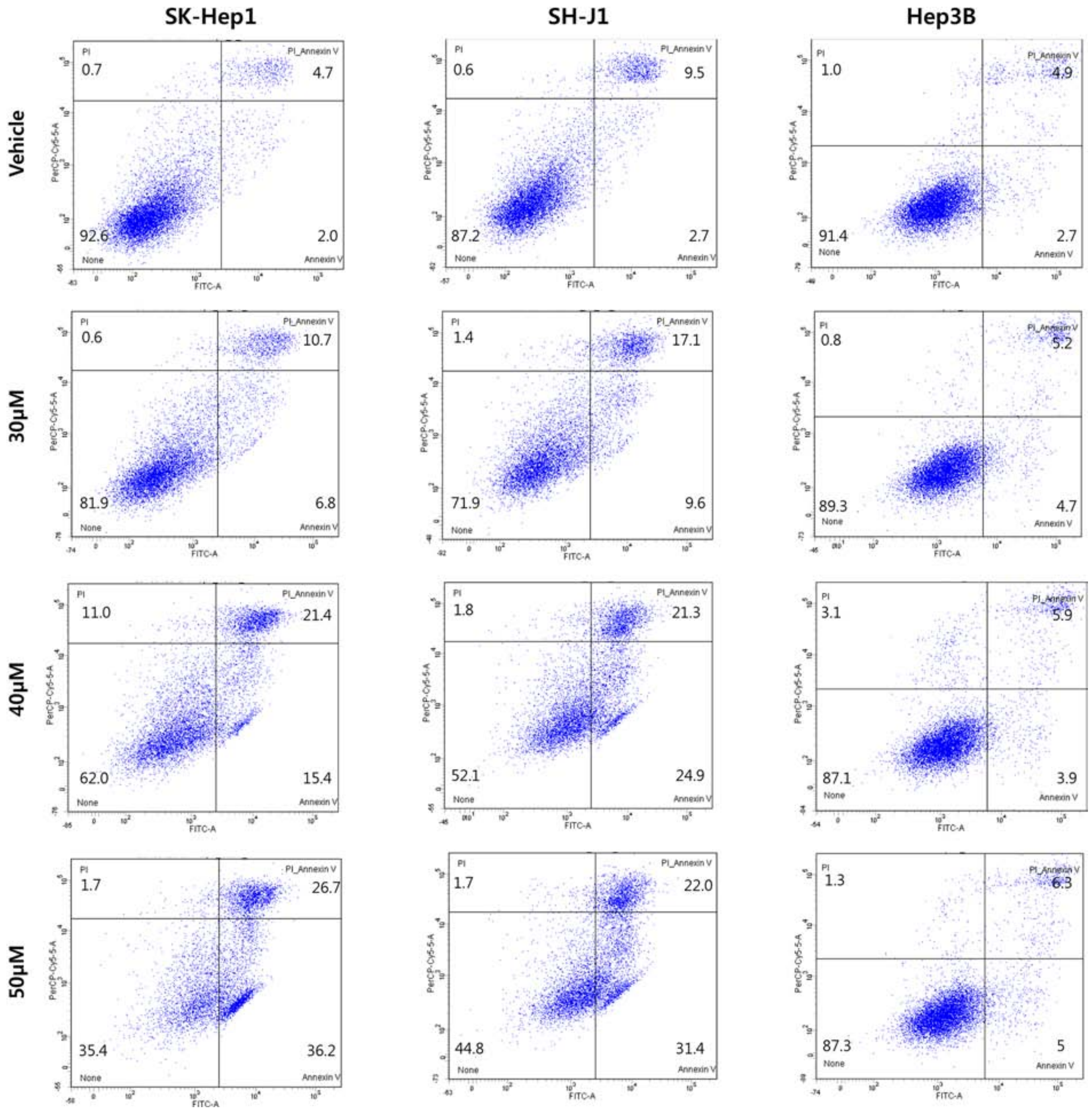

Supplementary Figure S5: Effect of luteolin on THLE-2 and HCC cell apoptosis. A. Concentration-dependent effects of luteolin on apoptosis were assessed using the PI and Annexin V double staining followed by flow cytometry 24 h after addition of luteolin.

Supplementary Table S1: Clinicopathological Characteristics of HCC patients

Clinicopathologic parameters	IHC *n* = 88
Age (range)	
<55 years	46
≥55 years	42
Gender (M/F)	
Male	61
Female	27
HBV	(-1)
Absent	22
Present	65
HCV	(-2)
Absent	77
Present	9
Tumor stage	
I	28
II	31
III	29
BCLC stage	
A	33
B	39
C	16
AFP	(-1)
<100 ng/ml	34
≥100 ng/ml	53
Vascular invasion	
Absent	33
Present	55
Tumor size	
≤3 cm	15
>3 cm	73
Edmondson grade	
I	14
II	38
III	36
Portal vein invasion	
Absent	73

(*Continued*)

Clinicopathologic parameters	IHC *n* = 88
Present	15
Median follow-up periods (range)	
Recurrence	23.47 months (1.15~134.30)
Overall survival	47.77 months (2.01~193.68)
Disease-free survival	23.47 months (1.15~134.30)

Supplementary Table S2: Relation between VRK1 protein levels and clinicopathological characteristics

Clinicopathologic parameters	VRK1 High (*n* = 7) IHC 2, 3	VRK1 Low (*n* = 81) IHC 0, 1	*P*	Clinicopathologic parameters	VRK1 High (*n* = 7) IHC 2, 3	VRK1 Low (*n* = 81) IHC 0, 1	*P*
**Age**			0.051	**AFP level**		(-1)	0.700
< 55 years	1	45		< 100 ng/mL	2	32	
≥ 55 years	6	36		≥ 100 ng/mL	5	48	
**Gender**			0.431	**Vascular invasion**			0.237
Male	6	55		Absent	1	32	
Female	1	26		Present	6	49	
**HBV**		(-1)	1	**Tumor number**			0.702
Absent	2	20		Single	5	49	
Present	5	60		Muliple	2	32	
**HCV**		(-2)	1	**Tumor size**			1
Absent	7	70		≤ 3 cm	1	14	
Present	0	9		> 3 cm	6	67	
**Liver cirrhosis**		(-3)	0.240	**Tumor size**			0.705
Absent	6	46		≤ 5 cm	4	38	
Present	1	32		> 5 cm	3	43	
**Tumor stage**			0.207	**Edmondson grade**			0.364
I	1	27		I	0	14	
II	5	26		II	5	33	
III	1	28		III-IV	2	34	
**BCLC stage**			1	**Portal Vein Invasion**			1
A	3	30		Absent	6	67	
B	3	36		Present	1	14	
C	1	15					

Supplementary Table S3: Primer sequences for real-time quantitative PCR of HCC cell lines

Genes		Sequences
CCND1	F	CAG AAC ACG GCT CAC GCT TAC CT
	R	CGG TGC AAC CAG AAA TGC ACA G
ACTIN	F	GTA CCA CTG GCA TCG TGA TGG A
	R	GCC ATC TCT TGC TCG AAG TCC AG

Supplementary Table S4: shRNA sequences targeting VRK1

C1	CCGGCCTGGTGTTGAAGATACGGAACTCGAGTTCCGTATCTTCAACACCAGGTTTTT
C2	CCGGGAAGTAAGGATGATGGCAAATCTCGAGATTTGCCATCATCCTTACTTCTTTTT
C3	CCGGGAGATATCAAGGCCTCAAATCCTCGAGGATTTGAGGCCTTGATATCTCTTTTTTG
C4	CCGGCGAGCATCGATGCACACAATGCTCGAGCATTGTGTGCATCGATGCTCGTTTTTTG
C5	CCGGGCGAGGTGGAAGTAATGATTACTCGAGTAATCATTACTTCCACCTCGCTTTTTG
